# Mitigating inequitable access to appropriate antibiotics in low- and middle-income countries

**DOI:** 10.1093/jacamr/dlaf061

**Published:** 2025-04-24

**Authors:** Idemudia Imonikhe Otaigbe

**Affiliations:** Department of Medical Microbiology, School of Basic Clinical Sciences, Benjamin Carson (Snr.) College of Health & Medical Sciences, Babcock University, Ilishan Remo, Ogun State, Nigeria

## Abstract

Access to effective medicines (e.g. antibiotics) is a fundamental human right. However, in contrast to high-income countries (HICs), many low- and middle-income countries (LMICs) lack appropriate and effective antibiotics. This is a paradox, and an inequitable scenario, as LMICs can have significantly higher burdens of infectious diseases than HICs and especially require appropriate antibiotics. Inequitable access to appropriate antibiotics results in patients being treated with substandard antibiotics, treatment failure, the emergence of antimicrobial resistance (AMR) and, inevitably, morbidity and mortality. Factors that hinder access to appropriate antibiotics in LMICs include: poor political will, weak health systems, complex bureaucratic protocols, poor implementation of National Action Plans on AMR, inadequate expertise in regulatory science, unfavourable macroeconomic policies and a poor investment climate. Clearly, multisectoral, collaborative approaches are required to effectively mitigate inequitable access to appropriate antibiotics in LMICs. Also, efforts (such as the African Medicines Regulatory Harmonization Initiative and the African Medicines Agency) to streamline bureaucratic processes and improve the registration and entry of appropriate antibiotics into LMICs are required. This review discusses factors responsible for inequitable access to appropriate antibiotics in LMICs, and makes recommendations to mitigate the problem. With rising rates of AMR, a dwindling antibiotic pipeline, and the dangers of a post-antibiotic era, it is clear that the time to act is now, as inequitable access to appropriate antibiotics in LMICs reduces the quality of healthcare, and threatens the achievement of Universal Health Coverage and, ultimately, the Sustainable Development Goals.

## Introduction

Access to effective medicines, such as antibiotics, is a fundamental human right and a key component of the Sustainable Development Goals.^1^ It is defined as ‘having drugs continuously available and affordable, at public or private health facilities, or drug outlets, that are within one hour’s walk of the population.’^1^ An analysis involving 101 countries, estimated, that adequate access to antibiotics, could annually reduce 75% of deaths (equivalent to 445,000 deaths), attributable to community-acquired pneumonia, in children younger than 5 years.^2^ However, access to appropriate antibiotics has yet to be achieved as a fundamental human right in many low- and middle-income countries (LMICs).^3,4^ Many LMICs, with high infectious disease burdens, lack appropriate and effective antibiotics.^5–7^ For example, a study conducted in Lao People's Democratic Republic (Laos), showed that only 29 out of 39 antibiotics in the WHO Access, Watch and Reserve (AWaRe) book for the classification of antibiotics were available in the country, and that no Reserve group antimicrobials were available.^8^ Similarly, another study of healthcare facilities in 20 LMICs (in Africa, Asia and the Caribbean) showed that many of the Access antibiotics were unavailable in most of the healthcare facilities surveyed in the study.^9^ In the absence of appropriate antibiotics, patients in LMICs are treated with antibiotics that ideally should be reserved as second- or third-line choices for antibiotic therapy.^10^ Another danger associated with poor access to appropriate antibiotics in LMICs is the use of substandard or less suitable antibiotics, resulting in suboptimal concentrations, treatment failure and, inevitably, considerable morbidity and mortality.^5–7,10–13^ For example, it is estimated that poor access to appropriate antibiotics results in about 5.7 million deaths each year.^7^ Sadly, most of these deaths occur predominantly among people living in poverty in LMICs.^7^ In addition to adverse clinical outcomes, patients in LMICs also experience adverse economic consequences (such as prolonged hospital stay and increased healthcare costs) when they are treated with inappropriate antibiotics.^6^

A lack of access to appropriate antibiotics, and the administration of substandard and/or inappropriate antibiotics, also drive the emergence of antimicrobial resistance (AMR).^12,14,15^ AMR is one of the biggest threats to global health, development and food security.^14,15^ Globally, AMR accounts for an estimated 700 000 deaths annually, and if unchecked is predicted to cause 10 million deaths annually worldwide by 2050.^16,17^ A sustained rise in AMR would also have devastating economic impacts, resulting in a 2% to 3.5% drop in global GDP by 2050, a massive loss of between 60 and 100 trillion US dollars in economic production, and widespread human misery.^16,17^ LMICs would be worse hit by a sustained rise in AMR, as it is predicted that about 4.1 million people in Africa will die by 2050 due to a sustained rise in AMR.^18^ The emergence of AMR has also resulted in many pharmaceutical companies being reluctant to invest in research and development (R&D) efforts for new and effective antibiotics.^19^ This is dangerous as a paucity of development in new and effective antibiotics and rising rates of AMR put the world in danger of a post-antibiotic era (i.e. an era characterized by a paucity of effective antibiotics and increased morbidity and mortality from severe and even minor infections).^20^ Individuals in LMICs would be worst hit in a post-antibiotic era due to high burdens of infectious diseases and weak health systems.^2,5,6,21^

Major responses to curbing AMR have focused on reducing both antibiotic consumption and inappropriate antibiotic use.^22^ Globally, a 46% rise (from 9.8 to 14.3 defined daily doses per 1000 population per day) in human antibiotic consumption has occurred over the past 20 years.^23^ However, these figures fail to paint the true picture of a lack of access to antibiotics in LMICs, since in several LMICs the problem is not excess antibiotics but a paucity of appropriate and effective antibiotics.^6,7,24^ Currently, more people in LMICs die from a lack of access to antibiotics than from AMR.^24^ The reality is that the 5.7 million annual deaths (most of which occur in LMICs) attributed to a lack of antibiotics far outweigh the estimated 700 000 annual deaths arising from AMR.^24^

Clearly it is imperative to mitigate the problem of poor access to appropriate antibiotics in LMICs. However, mitigating a problem involves the following steps. First is to clearly define the problem.^25,26^ Second is to identify factors that drive the problem.^25,26^ Finally it is necessary to proffer suitable recommendations or strategies to mitigate the problem.^25,26^ In this regard, this review will therefore follow suit, by clearly defining the terms ‘appropriate antibiotics’ and ‘access to appropriate antibiotics’. Next, factors that drive poor access to appropriate antibiotics are discussed. Finally, recommendations to improve access to appropriate antibiotics in LMICs are discussed.

## What is an appropriate antibiotic?

An appropriate antibiotic may be defined from the clinical (patient) or the population level. At the patient level, antibiotic use is considered appropriate when it is administered to a patient in full compliance with empirical antibiotic guidelines and/or antibiotic susceptibility test (AST) results.^5,6,16,27–38^ It implies that a patient who requires antibiotics, receives (or has access to) the right antibiotic, of the right quality (standard), at the right time, at the right dosage and for the right duration.^5,6,20,27–38^ In this regard, determining the appropriateness of an antibiotic, at the patient level, involves guidelines and AST results.^27–38^ However, many LMICs lack guidelines, antibiograms, effective antimicrobial stewardship (AMS) programmes and sufficient clinical microbiology services (CMS) to ensure the use of appropriate antibiotics at the patient or clinical level.^39–48^

At the population level, the term ‘appropriate antibiotic’ implies that a population has access to a spectrum of antibiotics that are capable of meeting the infectious disease needs of that population.^6,12^ For example, trachoma, a neglected tropical disease, affects rural populations (in Africa, Central and South America, Asia and the Middle East) with poor access to healthcare and water, sanitation and hygiene (WASH) facilities.^49^ Trachoma is the leading cause of blindness globally, and accounts for an estimated US$2.9 to 5.3 billion annual loss in productivity due to blindness and visual impairment.^50^ Efforts to curb the disease involve the SAFE (i.e. Surgery, Antibiotics, Facial cleanliness and Environmental improvement, including improved access to WASH facilities) strategy.^49^ An appropriate antibiotic required for the treatment of trachoma is azithromycin. However, the antibiotic is expensive and typically inaccessible for poor rural populations, affected with the disease.^49,50^ To improve access to azithromycin, the manufacturer has donated the antibiotic to elimination programmes (involving mass administration of azithromycin to affected populations), through the International Trachoma Initiative.^49,50^ This has made it possible for azithromycin to reach populations affected by the disease.^49,50^

Furthermore, determining the appropriateness of antibiotics at the population level requires population-level data on AMR patterns, antimicrobial consumption and pathogen dynamics.^6,12,39,51–54^ Such data will guide policy making, market forces (such as local antibiotic manufacturing, importation, supply, demand, etc.) and donor aid, to ensure a steady availability of appropriate antibiotics to a particular population.^6,12,39,52–54^ For example, data emanating from decades of multidisciplinary research were instrumental in guiding policy-making and donor aid to fight trachoma and make azithromycin accessible to populations affected with the disease.^53^ However, at the population level, many LMICs lack sufficient and accurate data on AMR, antibiotic consumption patterns and pathogen dynamics.^6,12,48,51–55^ As such, many LMICs import or locally manufacture antibiotics that are of no benefit to the infectious disease needs of their populations.^6,12^

## Defining access to appropriate antibiotics

Access to antibiotics may be viewed from several perspectives, as outlined below.

### General access to antibiotics

The first perspective refers to access to antibiotics (i.e. new or older antibiotics) in general,^1,6,12,54,55^ in a healthcare facility, community or nation.^1,6,12,50^ It implies that individuals or a population have access to antibiotics.^1,6,12^ However, because an antibiotic is available does not mean that it is appropriate for an individual or a population or equitably accessible to an individual or a population.^6,12,55,56^ For example, in many LMICs, where antibiotics can be easily obtained without a prescription (i.e. over-the-counter antibiotics) from an unlicensed medicine vendor, access to such antibiotics does not mean that the patient has received an appropriate antibiotic, particularly as many of these ‘antibiotics’ are substandard or falsified.^56^

### Access to appropriate antibiotics

The second perspective is more specific and refers to access to the appropriate antibiotic at an affordable price, at the right dosage, at the right time and for the required treatment duration.^6,12,50,55–60^ This perspective also includes access to good quality and effective antibiotics and entails the presence of strong governance mechanisms, political will and AMS practices to ensure the responsible use and sustained effectiveness of antibiotics.^6,12,50,56–60^ For example, many LMICs lack health insurance for their broad populations or health financing schemes, and patients have to pay out-of-pocket for healthcare, including antibiotics.^6,12,24^ Therefore, patients who cannot afford antibiotics are in essence denied access to appropriate antibiotics.^6,12,14,24^

### Access to clinically useful, older, generic, off-patent antibiotics

The third perspective expands the term ‘access to appropriate antibiotics’ to include access to older, generic, off-patent antibiotics that are still clinically useful and appropriate, in the management of several infectious diseases.^12,61–65^ For example, fosfomycin is an old generic off-patent antibiotic that is clinically effective in the treatment of MDR infections.^61^ Fosfomycin (and many clinically useful older generic off- patent antibiotics) are, however, unavailable in many LMICs due to a variety of factors.^12,61–65^ Some of these factors include: low economic incentives to manufacture and/or introduce these antibiotics into LMICs; a lack of inclusion of these antibiotics in antibiotic-prescribing guidelines; and low prescribing by physicians.^61–65^

### Access to new and effective antibiotics

The fourth perspective expands the term ‘access to appropriate antibiotics’ to include access to new and effective antibiotics.^6,12,66–68^ For example, a report by the Access to Medicine Foundation mentions cefiderocol and ceftazidime/avibactam as two new antibiotics indicated for the treatment of infections due to MDR Gram-negative bacteria.^12^ Importantly, MDR Gram-negative bacteria account for significant morbidity and mortality in LMICs.^48,52,67^ In this regard, a report by the WHO stated that 45% of deaths in Africa and South-East Asia were due to MDR bacteria and that strains of ESBL-producing *Klebsiella pneumoniae* were associated with elevated deaths in Africa and South-East Asia.^35,52^ However, access to these two (and other) new antibiotics in LMICs is poor.^12,67,68^

### All four perspectives of access to appropriate antibiotics are important in LMICs

It is important to have in view these four perspectives (i.e. a general access to antibiotics; access to appropriate antibiotics; access to clinically useful, older, generic, off-patent antibiotics; and access to new and effective antibiotics). This is due to the fact that access to antibiotics does not necessarily mean access to an appropriate antibiotic, or access to a new and effective antibiotic, or even to an older off-patent antibiotic (which may be urgently required by a patient or a population).^6,12^ Interestingly, in many LMICs the major problems lie with the second, third and fourth perspectives, or more specifically inequitable access to appropriate antibiotics.^6,12,49,50,55–58,61–68^

‘Inequity’ implies that individuals are denied quality healthcare (including appropriate antibiotics) due to a variety of demographic, geographical or socio-economic factors.^60^ In many LMICs, inequitable access to appropriate antibiotics is a fundamental problem.^6,12,49,50,55–58,61–68^ For example, the high costs of several antibiotics make them financially inaccessible to many patients in LMICs, who bear the costs of healthcare due to the lack of national health insurance programmes.^6,12,69^ In addition, logistic and infrastructure deficits, such as poor road networks, inefficient transport systems and weak supply chain management (SCM) systems, result in poor access to antibiotics in rural or remote communities in LMICs.^6,12,69^ Also, a paucity of antibiotic-prescribing guidelines, clinical microbiology services, antibiograms and AMS programmes results in inappropriate antibiotic prescribing, which ultimately denies patients from accessing the appropriate antibiotics they require.^45,69^

## Factors responsible for inequitable access to appropriate antibiotics in LMICs

Several factors are responsible for inequitable access to appropriate antibiotics in LMICs and are considered here.

### Dearth of political will by LMIC governments

Political will is ‘the commitment of political leaders and bureaucrats to undertake actions to achieve a set of objectives and to sustain the costs of those actions over time.’^70^ The demonstration of political will by an LMIC government is a key determinant in efforts to improve access to appropriate antibiotics in an LMIC.^5,6,12,71–74^ The presence of strong political will fosters a suitable regulatory and investment-friendly environment, required for the entry and/or local production of new and existing antibiotics.^5,6,12,71–73^ In addition, political will encourages donor agency support and funding for suitable or country-specific access strategies, which enable access to appropriate antibiotics.^5,6,12^ For example, the Ghanaian government, in partnership with the United Nations Industrial Development Organization (UNIDO), European Union, the German Development Corporation and other stakeholders, has created policies to boost local pharmaceutical production and also transform the nation into a pharmaceutical manufacturing hub in sub-Saharan Africa.^74^ However, many LMIC governments exhibit poor political will to improve access to antibiotics into their countries. This results in poor funding or support for access strategies, poor regulatory frameworks and an unsuitable investment climate.^5,6,12,39,75–77^

### Intractable bureaucratic protocols and poor regulatory frameworks

Major consequences of poor political will by LMIC governments are poor regulatory frameworks and intractable bureaucratic protocols, which hinder access to appropriate antibiotics by LMICs.^2,5–7,12,45,58^ For example, in several LMICs the registration of antibiotics is slow, costly, labour intensive, and characterized by a dearth of adequate technical capacity.^6,12,78–80^ In addition, pharmaceutical companies seeking to introduce their products into several LMIICs usually have to file individual applications to regulatory agencies in each country.^6,12,66^ These bureaucratic hurdles delay entry of appropriate antibiotics into LMICs, and also discourage pharmaceutical companies from initiating registration of antibiotics.^6,12,66^ Whereas some of these protocols are outdated and require review, another challenge faced by LMICs is a dearth of expertise in regulatory science.^6,12,78–83^ Regulatory science is defined as ‘the science that informs, facilitates and/or evaluates regulatory decision making.’^84^ Regulatory science is a multidisciplinary field that ensures the safety, efficacy and quality of products within regulated industries.^85^ Essentially, regulated industries are industries that are governed by specific regulations (including policies and standards) that apply from the product’s R&D stage, to product marketing and use.^85^ Examples of these industries include pharmaceuticals, medical devices, biotechnology, cosmetics and food.^85^ With regards to pharmaceutical industries, regulatory science focuses on the performance of medicine regulations and regulatory instruments; the development of tools and methods to back regulatory decision making; and the generation of evidence that informs regulatory decisions.^85,86^

The dearth of regulatory science expertise in LMICs arises from factors such as: limited training opportunities in regulatory science; poor incentives; inability of health agencies to attract and retain qualified and experienced staff; an absence of career structure; and job descriptions that do not sufficiently detail the required competencies for positions.^78–82^ As a result, most LMIC regulatory agencies have persistent human resource gaps, leading to perennial backlogs, lengthy review timelines, and challenges with registering antibiotics.^78–82^

Also, in several LMICs some regulations that are not necessarily targeted at the health sector largely influence the health sector and, ultimately, access to antibiotics.^87^ For example, in some LMICs, macroeconomic policies, such as stringent foreign exchange protocols, often deny importers the needed foreign exchange to procure products (e.g. antibiotics) from abroad and import them into their countries.^87^

Similarly, regulatory hurdles in high-income countries (HICs) also hinder the entry of appropriate antibiotics into LMICs.^66,88,89^ Some of these regulatory hurdles involve antibiotic R&D and approval of newly developed antibiotics.^66,89^ For example, a study showed that of 25 new antibiotics (introduced between 1999 and 2014) only 15 were launched in the country in which the originator is based.^66^ Also, only 12 of these antibiotics had registered sales in more than 10 countries.^66^ Consistent gaps between antibiotic development by pharmaceutical companies and registration of these newly developed antibiotics in many countries (particularly in LMICs with demand for new and effective antibiotics) represent an economic waste as they deny pharmaceutical companies access to new markets and significant return on investment.^88^

### Small market sizes in LMICs and low revenues from antibiotic sales

Since antibiotic development is a capital-intensive venture it usually results in newly introduced or existing antibiotics having high market prices.^6,12^ To ensure return on investment, pharmaceutical companies primarily focus on countries with significant market potential or large market sizes.^90^ Essentially, a nation’s market size for antibiotics (and other pharmaceutical products) is influenced by the nation’s population size, the government’s (or public) pharmaceutical expenditure per capita (public pharmaceutical expenditure per capita is defined by the WHO as ‘the public pharmaceutical expenditure divided by the number of inhabitants of the country’^91^) and the purchasing power of the populace.^90^ However, although the high burden of infectious diseases in LMICs creates a high demand for antibiotics, high levels of poverty, limited government expenditure on healthcare delivery, and country-specific socio-economic challenges reduce the purchasing power of both the government and the populace, and make new or existing antibiotics financially inaccessible to many individuals in LMICs.^6,12,90^ Therefore, many LMICs are unviable markets for the entry and distribution of new or existing antibiotics.^6,12,90^ However, some LMICs like Nigeria, Kenya, South Africa, Brazil, India and China present large market sizes, due to their large populations, larger patient numbers and purchasing power.^12^ As a result, these countries are investment destinations for large pharmaceutical companies.^12^

### Poor investment climate

Closely related to small market size is the poor investment climate in many LMICs.^12^ The term ‘investment climate’ refers to location- or country-specific factors (such as macroeconomic policies; regulatory, legal, financial and socio-political institutions; and infrastructure) that shape the opportunities and incentives for firms to invest productively, create jobs and grow.^92,93^ A conducive investment climate will attract investment, whereas a non-conducive investment climate will ultimately deter investment.^92,93^ Investment is the backbone of inclusive and sustainable growth, as it allows domestic and foreign investors to boost a nation's development.^92–94^

The importance of an investment climate is relevant for pharmaceutical companies who wish to introduce their antibiotics into LMICs, either through sales or local antibiotic manufacturing.^12,92–94^ However, many LMICs lack a suitable investment climate due to infrastructure deficits (e.g. unstable power supply, poor road networks, etc.), political instability and macroeconomic or fiscal policies that essentially do not encourage local or foreign direct investment in antibiotic production.^92–94^ For example, many investors, incur huge operating costs due to infrastructural deficits (e.g. unreliable electricity supply), insecurity and corruption.^92–94^ Also, a poor investment climate and unfavourable economic policies have resulted in the exit of pharmaceutical manufacturers from some LMICs.^12,95^

### Dearth of local antibiotic manufacturing

One powerful way to ensure access to appropriate antibiotics in LMICs is to encourage local antibiotic production.^6,12,96^ According to the 71st World Health Assembly in 2021, the ‘integration of local production into overall health systems strengthening can contribute to sustainable access to quality-assured, safe, effective and affordable medicines and other health technologies, and can help to prevent or address medical product shortages, achieving universal health coverage and strengthening of national health emergency preparedness and response and minimizing public health hazards, recognizing also that local production can contribute to other national development goals, such as catalysing local capacity in innovation, strengthening human capital and expertise and building a knowledge-based economy.’^96^ In this regard, the benefit of local antibiotic production expands beyond health, as it not only ensures a local supply of antibiotics, but has related socio-economic benefits of creating jobs and reducing poverty, both of which have positive impacts on population health and economic development.^97–99^ In addition, local antibiotic production makes antibiotics available at lower costs than when imported.^97–99^ For example, an analysis by McKinsey and Company showed that costs of locally manufactured pharmaceutical products in Ethiopia and Nigeria were about 5% to 15% lower than imported pharmaceutical products.^97^ In spite of these benefits (of local antibiotic manufacturing) there is a dearth of local antibiotic manufacturing in LMICs due to a variety of reasons, including: a poor investment climate; poor political will; a dearth of technical expertise; weak regulatory frameworks; and inadequate access to finance such as loans.^6,12,97,98^

However, some LMICs are major producers of antibiotics.^6,12,97–99^ For example, manufacturing of active pharmaceutical ingredients (APIs) occurs predominantly in China and India, with about 40% of global API production occurring in China.^12,100,101^ Similarly, it is estimated that about 80% of pharmaceutical manufacturing in Africa occurs in Egypt, Morocco, Algeria, Tunisia, Ghana, Nigeria, Kenya and South Africa.^99^ However, pharmaceutical manufacturing in Africa still lags behind India and China, and is insufficient to meet the needs of Africa’s population of 1.3 billion people.^97–99^ In addition, Africa still imports over 70% of its pharmaceutical products.^97^ Also, Africa has limited API and R&D capacities.^99^ The majority of Africa’s pharmaceutical manufacturing is focused on generic products (representing about 70% of the total local production value) and downstream processes of the manufacturing value chain (such as formulation or finished product manufacturing and packaging).^98,99^ Pharmaceutical manufacturing also occurs in most countries in Latin America and the Caribbean. Some of these pharmaceutical products are exported, although all countries in the region import pharmaceutical products.^102^ For example, pharmaceutical manufacturing in Mexico, Brazil and Peru meets 46%, 35% and 12% of domestic demand for pharmaceutical products, respectively.^102^ Also, the majority of the medicines produced in the region are generics and these are mainly sold to domestic and regional markets.^102^

There are, however, concerns about the quality of manufacturing processes and adherence to good manufacturing practice (i.e. GMP, which is defined as a system for ensuring that products are consistently produced and controlled according to quality standards^103^) by some manufacturers in LMICs.^104^ However, with strong regulatory frameworks and a suitable investment climate, local pharmaceutical manufacturing has the potential to improve access to quality-assured medicines (such as appropriate antibiotics) and other health technologies; achieve universal health coverage; and ultimately attain the health-related targets and broader developmental objectives of the Sustainable Development Goals.^105^

### Underutilization and inadequate funding of evidence-based access strategies

There are clear-cut evidence-based access strategies that have the potential to increase access to appropriate antibiotics in LMICs.^12^ However, a recent report by the Access to Medicine Foundation has shown that several pharmaceutical companies and other key stakeholders (such as LMIC governments, donor agencies, etc.) in the antibiotic landscape of several LMICs are underutilizing these access strategies.^12^ For example, as discussed above, local antibiotic manufacturing is a major access strategy.^6,12,97–99,105^ Other examples of these strategies (some are discussed in the ‘Recommendations’ section below) include: making antibiotics more affordable to LMIC populations through innovative pricing strategies, and participating in pooled procurement of antibiotics.^12^ Clearly, the use of these access strategies will improve access to appropriate antibiotics in LMICs.^12^

A related problem is the inadequate funding of access strategies by donor agencies and HICs.^27^ Ensuring access to appropriate antibiotics in LMICs requires significant funding.^27^ However, funding from donor agencies and HICs to support access strategies, though commendable, still remains inadequate.^27,49,50,54^ As mentioned earlier, new and even existing antibiotics are unaffordable to many individuals in LMICs.^12^ Therefore, in the absence of funding, direly needed antibiotics will continue to be unavailable to patients in LMICs.^12,27,49,50,54,57^

### Fragmented approaches to improve access to antibiotics in LMICs

A major problem with efforts to improve access to appropriate antibiotics in LMICs is the fragmented nature of these efforts by donor agencies, HICs, LMIC governments, pharmaceutical companies and other key stakeholders.^27,50,106–108^ Fragmentation undermines the effectiveness of health programmes, debars sustainability, overburdens (rather than strengthens) the fragile health systems of LMICs and ultimately threatens attainment of the health-related Sustainable Development Goals.^106–108^ Drivers of fragmentation include: divergent interests (among stakeholders); inappropriate policies; siloed programmes that are not integrated into national health systems; and non–evidence-based, politically motivated interventions or programmes.^27,106,107^ In addition, fragmentation results in efforts that are not context specific, as a strategy that works in one particular LMIC may fail woefully in another LMIC.^106–108^ The complex interplay of socio-cultural, political and economic dynamics, which hinders access to appropriate antibiotics both within and across LMICs, is diverse and requires clear-cut holistic and collaborative approaches (involving multi-stakeholder strategic partnerships).^106–108^ Therefore, efforts to improve access to appropriate antibiotics in LMICs may not yield desired outcomes in the absence of pooled, collaborative efforts specific to the LMIC context by all stakeholders.^12,106–108^

### Waning R&D efforts regarding new and effective antibiotics

Waning R&D efforts regarding new antibiotics are a major barrier to access appropriate antibiotics, both in LMICs and globally.^6,12^ Essentially, the majority of antibiotic R&D occurs in HICs (although some LMICs such as India, South Africa and Brazil are involved in antibiotic R&D).^109–112^ For example, the Antimicrobial Drug Discovery Hub is an international collaboration between South Africa and the UK to discover new antibiotics.^109^ Many HICs have considerably lower burdens of infectious diseases (and therefore lower demand for antibiotics) than LMICs.^6,12^ Consequently, with declining revenues from antibiotic R&D and high burdens of non-communicable diseases, several pharmaceutical companies in HICs are exiting antibiotic R&D and transitioning to R&D for drugs and therapeutics for non-communicable diseases.^6^ This economic transition is potentially disastrous for LMICs, which have high burdens of infectious diseases, weak health systems, and minimal or no capacity for local antibiotic R&D.^6^ Also, such a transition means that LMICs would eventually have no potent antibiotics to fight infectious diseases, resulting in high rates of morbidity and mortality.^6^ Investment in R&D for new antibiotics is characterized by a low return on investment and market failures due to a variety of reasons.^89^ Firstly, antibiotic development is capital intensive and time consuming, as it takes about 10 to 15 years to develop an antibiotic and fulfil regulatory requirements for approval and licensing.^89,113^ Such regulatory requirements ultimately delay the market entry of new antibiotics.^12,89,113^ Secondly, there are significant challenges during antibiotic development (e.g. difficulties in discovering novel chemical structures that are safe for human consumption, identifying target genes or receptors for new drug candidates, etc.) that result in high failure rates during preclinical and clinical phases of R&D for new antibiotics.^89,113,114^ For example, a success rate of only 1.5% to 3.5% is associated with developing an antibiotic from the preclinical research stage to the stage of achieving regulatory approval.^89,114^ This high failure rate poses a significant economic challenge as it makes investment in antibiotic preclinical and early-stage clinical research a high-risk venture.^89^ It is therefore no surprise that many potential investors shy away from investing in these key phases of antibiotic R&D.^89^ As a result, new classes of antibiotics are a rare occurrence in the antibiotic pipeline and instead many antibiotic developers focus on minor modifications to pre-existing classes of antibiotics.^89^ Thirdly, few incentives exist to support and cushion the potential risks associated with antibiotic R&D.^89^ Fourthly, in spite of the capital-intensive nature of antibiotic R&D, market entry of antibiotics typically involves moderate pricing strategies that are designed to ensure affordability and accessibility to patients who require them.^12,89^ Such moderate pricing strategies typically put newly introduced antibiotics at an economic disadvantage since the profit margin between the unit cost of production and sales is very minimal.^12,89^ Fifthly, sales volumes for newly introduced antibiotics are low since newly introduced antibiotics are typically used as last-resort antibiotics when older existing antibiotics fail to treat critical infections.^89,115^ In addition, ongoing efforts to improve appropriate antibiotic use can dissuade physicians from readily prescribing new antibiotics, especially if older existing antibiotics are still effective.^89^ This results in low demand and low sales volumes for new antibiotics.^89^ However, as mentioned earlier, LMICs are also challenged by poor access to clinically useful, older, generic, off-patent antibiotics that are no longer being produced or supplied by pharmaceutical companies, due to a lack of expected profit and a lack of awareness of or misunderstood demand.^6^ Sixthly, unlike drugs for non-communicable diseases (such as hypertension, asthma and diabetes mellitus), antibiotics are typically used for short durations.^89^ This again is an economic disadvantage for investors, as typically a mix of appropriate pricing strategies, high sales volumes and long-term market demand drive high return on investment for any good or service.^89^ Therefore, short durations of treatment typically translate to low sales volumes, in contrast to the longer treatment durations for drugs for non-communicable diseases.^89^ Finally, the emergence of resistance to a new antibiotic essentially renders such an antibiotic useless and a market failure.^89^

### Weak health systems in LMICs

Sustainable Development Goal 3 includes access to quality essential healthcare services; access to safe, effective, quality, and affordable essential medicines and vaccines for all; fighting communicable diseases; achieving universal health coverage and health financing.^59^ There is a clear association between access to quality healthcare services and access to appropriate antibiotics.^59^ If people cannot access quality healthcare services, then this significantly reduces their opportunity of accessing appropriate antibiotics.^6,43^ However, poor access to quality healthcare is a perennial problem in several LMICs.^6,43^ In simple terms, the health systems of many LMICs lack the capacity to deliver quality healthcare, including guaranteeing access to appropriate antibiotics, to patients.^6,43,116^ Although health systems in LMICs are overwhelmed by a myriad of problems,^6,43^ this review focuses on those challenges that pertinently hinder access to appropriate antibiotics in LMICs.

- **Poor geographical access to quality healthcare services:** Many rural areas in LMICs lack effective health facilities, as most health facilities are located in urban areas.^43^ Clearly, an inability to access healthcare prohibits patients from accessing appropriate antibiotics.^6^ It is therefore not uncommon for patients in rural areas to visit unregulated and unlicensed vendors or facilities to access healthcare or purchase medicines (including antibiotics).^6,117^ For example studies conducted in rural parts of Ghana, India and Brazil showed that poor geographical access to health services was associated with purchase of antibiotics from unlicensed vendors and inappropriate antibiotic use.^117–119^ However, the quality of antibiotics obtained from these unlicensed vendors or facilities is usually substandard or even falsified.^8,56,120,121^
- **Poor financial access:** The problem of poor geographical access to health services in LMICs is compounded by a lack of health financing and national health insurance programmes that guarantee access to care as a fundamental human right.^6,43,59^ Poverty and unemployment are prevalent challenges in LMICs.^6,43,59^ Therefore, in the absence of health financing schemes (e.g. national health insurance programmes), healthcare is received as a privilege based on an individual’s ability to pay rather than as a fundamental human right.^4,6,43,59^ Since access to health services in many LMICs is out-of-pocket, people who can’t afford to pay for health services are ultimately denied access to healthcare, including appropriate antibiotics.^6,43^ For example, studies done in Nigeria,^122^ Kenya,^123^ Nepal^124^ and Paraguay^125^ showed that inability to pay for healthcare was a major reason why people did not visit a health facility when ill.
- **Limited progress in the implementation of National Action Plans on AMR in LMICs:** In 2015 the WHO adopted the Global Action Plan on AMR.^54^ The Action Plan spells out the need for countries to develop National Action Plans (NAPs) to combat AMR.^54^ In addition, the Action Plan emphasizes the need to ensure both access to and judicious use of appropriate antibiotics.^54^ Although several LMICs have developed NAPs for AMR,^126^ implementation of these plans remains poor due to a variety of reasons.^39^ A major reason is insufficient political will to implement these plans.^39^ In addition, implementation is stalled by a paucity of funds, poor multisectoral coordination, and defective or absent surveillance systems and a lack of technical capacity.^39^ The absence of functional NAPs on AMR hinders LMIC governments from formulating and implementing clear-cut policies that would identify country-specific antibiotic requirements, and ensure access to such antibiotics.^39,75–77^
- **Paucity of effective AMS programmes:** A problem closely related to the limited implementation of NAPs on AMR is the paucity of effective AMS programmes in LMICs.^22^ An AMS programme is defined as ‘an organizational or system-wide health-care strategy to promote appropriate use of antimicrobials through the implementation of evidence-based interventions.’^22^ In particular, the WHO recommends AMS programmes as an effective intervention for curbing inappropriate antibiotic use and AMR in healthcare facilities in LMICs.^22^ Effective AMS programmes also ensure equitable and sustainable access to effective antimicrobials, and improve treatment outcomes of infectious diseases.^22^ However, the implementation of AMS programmes in LMICs is quite low due to a paucity of funds, lack of political will and lack of technical expertise.^5,22,46–48^ Notably, a lack of AMS programmes drives inappropriate antibiotic prescribing practices and inadequate access to appropriate antibiotics, as patients will typically consume antibiotics based on the inappropriate prescriptions they have received.^5,22,46–48^ In addition, some funders may request that antibiotic developers should have AMS plans incorporated into their funding proposals to ensure continued efficacy of novel antibiotics.^127^ For example, the Combating Antibiotic-Resistant Bacteria Biopharmaceutical Accelerator (i.e. CARB-X: a global non-profit partnership whose overall goal is to accelerate the development of novel antibiotics for MDR bacterial infections) has developed a guide to support CARB-X-funded developers in drawing up stewardship and access plans for novel antibiotics.^127^ Such plans are expected to ensure responsible stewardship and appropriate access to novel antibiotics for LMICs.^127^
- **Poor access to quality clinical microbiology services:** Clinical microbiology services (CMS) provide quality diagnostic microbiology services, clinical consultation (such as guidance on appropriate specimen selection, collection, transport and processing; reporting test results accurately and clearly; interpreting test and antimicrobial susceptibility results; providing advice on the management of patients with infectious diseases; and providing advice on appropriate antibiotic prescribing), education (training residents; providing education to health workers on sample collection and transport; AMS, etc.) and research.^40^ The involvement of clinical microbiologists in the diagnostic/antibiotic prescription pathway ensures synergy between clinical, epidemiological and laboratory data.^40,45^ Such synergy ensures antibiotic therapy is guided to suit the clinico-epidemiological characteristics of each patient.^40,45^ This not only enables appropriate antibiotic therapy but also reduces the occurrence of adverse drug effects and improves patient outcomes.^40^ In addition, effective CMS are integral in the development of antibiograms.^45^ These antibiograms inform the development of local antibiotic guidelines, guide the deployment of appropriate empirical antimicrobial therapy in patient care, and ultimately improve patient outcomes.^45,51^ However, many LMICs lack access to quality CMS.^40–42,44,45^ Poor access to quality CMS in many LMICs may be viewed from two perspectives: inadequate access and inequitable access.^5,40–42,44,45^ Inadequate access to CMS connotes an insufficient quantity and quality of CMS, whereas inequitable access to CMS refers to an uneven distribution of quality CMS in a given LMIC.^5,40–42,44,45^ For example, laboratories in many LMICs are usually located in urban areas.^5,44^ Managing infectious diseases and prescribing antibiotics without the guidance of CMS results in poor access to appropriate antibiotic therapy and ultimately poor clinical outcomes.^5,40–42,44,45^
- **Underutilization of CMS by clinicians:** The dearth of quality CMS is exacerbated by the poor utilization of available CMS by clinicians in many LMICs, as they assume that the guidance of CMS is not necessary in the management of infections or in the choice of appropriate antibiotic therapy.^5,128,129^ This is due to the fact that clinicians in LMICs may have a poor appreciation of the need to use CMS in guiding antibiotic prescriptions.^5,128,129^ Also, many clinicians in LMICs have a poor understanding of the complex relationship between underutilization of CMS, inappropriate antibiotic use, and the eventual emergence of AMR and poor clinical outcomes.^5,128,129^ In addition, some clinicians are aware of the deficient quality of laboratory services in LMICs and therefore doubt the integrity of laboratory results and subsequently withhold the integration of these results into clinical decision-making.^5,128,129^ The long turnaround times associated with culture and antimicrobial susceptibility results also discourages some clinicians from using CMS as it is believed (by some clinicians) that by the time CMS laboratory reports are eventually released they are no longer useful in patient management.^5,128,129^ Clearly, the underutilization of CMS results in inappropriate antibiotic use and poor clinical outcomes.^5,128,129^
- **Poor surveillance structures, inadequate data and poor demand forecasting:** Many LMICs lack effective surveillance structures and data to track patterns of AMR, antibiotic consumption and the efficacy of existing antibiotics.^5,6,23,44,45,52^ For example, a study conducted in 14 African countries showed that less than 1.3% of the 50 000 medical laboratories (forming the laboratory networks of the 14 participating countries) had the capacity to evaluate AMR.^52^ Also, the study showed that of 187 000 samples tested for AMR, 88% did not include records of patients’ clinical profiles (such as diagnosis, site of infection, comorbidities and previous antimicrobial usage) while the remaining 12% had incomplete information.^52^ The absence of data on resistance patterns and antimicrobial consumption results in poor demand forecasting (i.e. the inability of policy makers and pharmaceutical companies to accurately identify unmet needs for specific antibiotics and develop strategies that would improve access to such antibiotics).^12,52^
- **Poor procurement processes and supply chain management systems:** The word ‘procurement’ is described as ‘the overall process of acquiring goods, civil works and services. This includes all functions from the identification of needs, selection and solicitation of sources, preparation and award of contract, and all phases of contract administration through the end of a services’ contract or the useful life of an asset.’^130^ The procurement process may also be defined as ‘the process that starts with the identification of a need and continues through planning, preparation of specifications/requirements, budget considerations, selection, contract award, and contract management. It ends on the last day of the warranty period.’^131^ Clearly, these definitions highlight the fact that the procurement process is a very detailed and organized process.^130,131^ A critical component of the procurement process is demand forecasting, which is described as ‘the ongoing process of projecting which products will be purchased, where, when, in what quantities and by whom.’^132^ Demand forecasting is instrumental in fulfilling five major roles in ensuring access to appropriate antibiotics.^132^ Firstly, it provides a clear picture of demand (or unmet needs) for antibiotics and therefore ensures that the procurement (and supply) process addresses this demand.^132^ Secondly, it guides effective planning and execution of the procurement process.^132^ Thirdly, by identifying unmet needs or demands for particular antibiotics, it facilitates R&D of new antibiotics, or sustained manufacture or production of existing antibiotics that are capable of meeting these demands.^132^ Fourthly, the clear delineation of unmet needs for particular antibiotics guides advocacy (by both government and non-governmental or civil society actors) for increased political commitment to address unmet needs for appropriate antibiotics.^132^ Fifthly, it strengthens the ability of the procurement process and the supply chain to ensure timely and sustained delivery of appropriate antibiotics to patients.^132^ However, successful demand forecasting requires adequate data (e.g. antimicrobial consumption and resistance patterns).^12,52,132^ As mentioned earlier, the health systems in many LMICs are challenged by poor surveillance structures and inadequate data, resulting in poor demand forecasting and inefficient procurement processes.^12,23,44,45,51^ Inefficient procurement processes subsequently lead to antibiotic shortages and deny citizens in LMICs access to appropriate antibiotics.^6,12^
  Besides lacking efficient procurement systems, LMICs lack efficient supply chain management (SCM) systems, which guarantee access to appropriate antibiotics to patients.^133^ SCM refers to ‘the activities needed to provide a final product or service, which involves the coordination of activities, starting with the raw materials and ending with the delivery of the final product or service to the end customer. At the end of the product’s or asset’s usable life, supply chains are also responsible for coordinating the recycling, remanufacturing (i.e. the rebuilding of a product to its original specifications using reused parts), or disposal of the final product.’^133^ Health systems require efficient SCM systems, which ensure that health products and/or services are available to the health system (healthcare staff, patients, etc.) at the right place, price, time, cost, quality, efficacy and quantity.^133^ Whereas procurement seeks to acquire health products and services (such as appropriate antibiotics), effective SCM facilitates the timely delivery of appropriate antibiotics to patients.^12,131–133^ However, the health systems of many LMICs lack effective procurement and SCM systems, required for sustained access to appropriate antibiotics.^6,12^ This results in shortages of appropriate antibiotics and the use of alternative (and often inappropriate antibiotics) in patient care, resulting in poor clinical outcomes, increased healthcare costs (borne by patients) and the emergence of AMR.^6,12,134^ For example, cefazolin is an antibiotic recommended for use in surgical antibiotic prophylaxis.^135,136^ However, there is poor compliance with these recommendations in many LMICs.^137–139^ Instead, broad-spectrum antibiotics, such as third-generation cephalosporins, are used for surgical antibiotic prophylaxis.^137–139^ A major reason for the poor compliance with guidelines is the unavailability of cefazolin in many LMICs.^134,140^ In this regard, a study done in Ethiopia showed shortages of cefazolin in Ethiopia’s health system (due to challenges with procurement and supply of the antibiotic) and the subsequent use of broad-spectrum antibiotics as alternatives.^141^ Poor compliance with guidelines for surgical antibiotic prophylaxis is associated with adverse clinical (such as the occurrence of surgical site infections, increased length of hospitalization, morbidity and the emergence of AMR) and economic (such as increased costs of healthcare borne by patients) outcomes.^136–141^
- **Substandard and falsified antibiotics:** Clearly, a significant drawback of ineffective procurement processes and SCM systems is a shortage of appropriate antibiotics, and the subsequent procurement, distribution and use of inappropriate antibiotics in health systems in LMICs.^12^ However, a significant proportion of these inappropriate antibiotics are substandard or falsified antibiotics.^12,142^ Substandard or ‘out-of-specification’ medicines are authorized medical products that fail to meet either their quality standards or their specifications, or both.^13^ Compromise in the quality of medicines, including antibiotics, may occur during manufacture, storage, distribution, or at the point of compounding and dispensing.^98,104,143^ Falsified medicines are defined by WHO as ‘medical products that deliberately/fraudulently misrepresent their identity, composition or source.’^13^
  Globally, about 28% of falsified medicines are antibiotics.^10,120^ Also, between 2014 and 2016, antibiotics accounted for 36% of falsified medicines seized by customs worldwide.^144^ According to the WHO, about 17% of substandard or falsified medicines in LMICs are antibiotics.^10,145^ In addition, an estimated US$30.5 billion is spent on substandard and falsified medicines in LMICs, accounting for 10.5% of medicine samples in the supply chain of these countries.^10^ The administration of substandard or falsified antibiotics is an example of inappropriate antibiotic therapy and a major barrier to access to appropriate antibiotics in LMICs.^5,6^ When substandard or falsified antibiotics are administered to patients, they result in treatment failure, poor clinical outcomes and the emergence of AMR.^5,6,121^

## Recommendations to improve access to appropriate antibiotics in LMICs

### Intensify global, regional and national dialogues to improve access to appropriate antibiotics in LMICs

The ongoing dialogues and forums regarding access to appropriate antibiotics in LMICs need to be intensified, as there is a need to continually draw attention (at global, regional and national levels) to the dangers of inadequate access to appropriate antibiotics in LMICs.^71–73,75–77^ In this regard, it is crucial to intensify dialogues and stakeholder engagements (at global, regional and national levels) to improve access to appropriate antibiotics in LMICs.^71–73,75–77^ For example, an important outcome of the second High Level Meeting on AMR, held at the 79th Session of the United Nations General Assembly (UNGA 79), in September 2024, was the approval of a Political Declaration by UN member states that establishes clear targets to reduce AMR-related deaths, promote sustainable financing, and expand the number of countries with funded NAPs on AMR.^71^ In particular, the problem of equitable access to antibiotics in LMICs was addressed in the political declaration.^71^ Also, a side event at the UNGA 79 entitled ‘Sustainable Access to Effective Antibiotics: The Path from UNGA to IMPACT’ focused on the issue of access to antibiotics in LMICs.^72^ Such forums should be encouraged as they are instrumental in drawing the attention of policy-makers and decision-makers (to the problem of access to appropriate antibiotics in LMICs) and therefore increase opportunities for public and health policy reforms to improve access to appropriate antibiotics in LMICs.^71–73,75–77^

### Improve political will

In addition to the above mentioned dialogues and forums, a priority recommendation is to build political will to improve access to appropriate antibiotics in LMICs.^71–73,75–77^ In the absence of strong political will by LMIC governments, such dialogues and forums are in vain.^71–73,75–77^ Building political will is a sum of three elements: political want (i.e. LMIC governments are keenly interested in making appropriate antibiotics more accessible to their nations); political can (i.e. LMIC governments possess the required technical, regulatory, infrastructural and financial capacity to improve access to appropriate antibiotics in their countries); and political must (LMIC governments consider it imperative to improve access to appropriate antibiotics in their countries).^146^ Achieving this equation (i.e. the sum of political want, political can and political must) will, however, involve the following policy recommendations.

The first recommendation regards the role of LMIC governments.^27,30,72,73,75,76,106–108,147^ In demonstrating political will, or commitment, to improve access to appropriate antibiotics, LMIC governments must ensure that all stakeholders in the nation’s healthcare delivery landscape are regulated and work in a holistic manner.^27,30,72,73,75,76,106–108,147^ In this regard, LMIC governments must exercise the required leadership and ensure that initiatives (such as initiatives funded by donor organizations or multinational pharmaceutical companies) to improve access to appropriate antibiotics do not occur in isolation but are: ‘country owned’; integrated into other ongoing health system programmes in the nation; and strengthen the nation’s health system.^27,30,72,73,75,76,106–108,147^ The term ‘country owned’ or ‘country ownership’ implies that LMIC governments exercise their regulatory roles to ensure that donor-funded (or pharmaceutical company–funded) initiatives (to improve access to appropriate antibiotics) are conceptualized, formulated, implemented, monitored and evaluated in accord with national development strategies or health policies.^147–152^ Also ‘country ownership’ means that LMIC governments provide the required leadership and coordination of dialogues and stakeholder engagements (such as with donors, HICs, pharmaceutical companies, civil societies, professional bodies, the private sector and other key stakeholders in the LMIC’s AMR landscape) to ensure such initiatives are result oriented and ultimately succeed.^147–152^ For example, the World Bank^148^ states: ‘One of the most important services the Bank can provide is to ensure that the process of policy reform is “internalized” in the country, as quickly as possible, so that the reform program, is designed by the country itself and integrated into its long-term development program.’^148^ Similarly, country ownership and coordination of donor-funded programmes (by LMIC governments) has been associated with increased effectiveness of such programmes.^153^

Similarly, the second recommendation emphasizes the need for LMIC ministries of health to fully exercise their regulatory functions and ensure that donor-funded (or pharmaceutical company–funded) initiatives (to improve access to appropriate antibiotics) are country specific (i.e. take into consideration the antibiotic needs and the socio-economic, political and population health peculiarities of the nation) and strengthen the nation’s health system.^27,30,39,73,75,76,147^ For example, the WHO, Asia-Europe Foundation and other partners are working with the Indonesian Ministry of Health to develop an Indonesia-specific health sector action plan, based on the WHO’s people centred approach, to address AMR in human health.^154,155^ The WHO people-centred approach to address AMR in human health is a core package of interventions to support NAPs on AMR.^154,155^ A major aspect of the approach is to ensure equitable access to quality-assured antimicrobials through adequate forecasting, procurement and distribution mechanisms. Also, the approach seeks to remove financial barriers to accessing antimicrobials by deploying appropriate pricing strategies such as health financing/insurance schemes.^154,155^ In this regard, the Indonesia-specific health sector action plan will be integrated into Indonesia’s national health transformation agenda, (including efforts to strengthen primary healthcare in the country; build health system resilience; and emergency preparedness and response capacities) and will complement ongoing One Health activities to address AMR at the human–animal–environment interface.^155^

The third recommendation is that the process of building political will requires taking advantage of ‘windows of opportunity’ in an LMIC’s political climate.^146,156,157^ These ‘windows of opportunity’ are referred to as ‘policy windows’.^156,157^ Policy windows are described as ‘exceptional, fleeting periods of time when there is a greater likelihood of initiating policy change than usual’.^157^ Policy windows create an opportunity for key stakeholders (who are interested in building political will) to bring matters to the agenda of government with a view to influencing policy reforms.^75,76,146,156,157^ For example, the entry of a new government with a keen interest in making policy reforms is an example of a policy window.^156–158^ Such a government would be more likely to listen to stakeholders and implement reforms to improve access to appropriate antibiotics.^156–158^

The fourth recommendation regards the benefit of including individuals (referred to as ‘policy entrepreneurs’) in efforts to build political will to improve access to appropriate antibiotics in LMICs.^157,159–161^ Policy entrepreneurs are individuals who actively engage and collaborate in efforts to promote reforms or innovations in national policy and decision making.^157,159–161^ Policy entrepreneurs may be in or out of government, but they are adept at advocacy, building strategic alliances and framing issues in a manner that makes them more likely to gain the support or approval of an incumbent LMIC government.^157,159–161^ For example, in some LMICs top government functionaries and individuals outside government have spearheaded health reforms, including reforms to improve access to appropriate antibiotics.^158,159,162,163^

A fifth recommendation is the inclusion of civil society organizations in efforts to build political will to improve access to appropriate antimicrobials in LMICs.^163–166^ This recommendation is in line with recommendations by the Interagency Coordination Group on AMR to engage (at global, regional, national and local levels) civil society organizations as key stakeholders in the One Health response to AMR.^164^ Civil society organizations are instrumental in efforts to build political will as their public advocacy and lobbying serves to constantly bring issues to the agenda of governments, pharmaceutical companies and global organizations.^163–166^

### Build strategic partnerships to improve access to appropriate antibiotics

Beyond dialogues and efforts to improve political will is the need for clear-cut strategies and actions to improve access to appropriate antibiotics in LMICs.^75,76^ Clearly, several LMIC governments have expressed both ‘political want’ and ‘political must’ to improve access to appropriate antibiotics in their respective countries. However, the major gap is in the domain of ‘political can’ as many LMICs lack the needed technical, financial and infrastructural capacity to take the steps required to improve access to appropriate antibiotics.^39,75,76^

For example, developing and implementing NAPs on AMR involves huge costs, which many LMICs cannot afford.^5,39,73,75,76^ To bridge these gaps (i.e. funding, technical, infrastructural gaps, etc.), LMIC governments can benefit from strategic partnerships with other LMIC governments, pharmaceutical companies (multinational and local), HICs and global/donor organizations.^39,73,75,76^ For example, a major lesson from the COVID-19 pandemic is the power of partnerships in combating issues.^167–169^ Through formidable partnerships, LMIC governments were able to deploy and scale up artificial intelligence and molecular platforms to facilitate diagnosis, management, vaccine delivery and public health decision-making during the pandemic.^167–169^ Lessons learnt from such partnerships should be applied to efforts to improve access to appropriate antibiotics in LMICs.^57,170–172^ Essentially, strategic partnerships can support LMIC governments to provide the required technical expertise, funding and infrastructure required to improve access to appropriate antibiotics.^68,173,174^ To achieve maximum impact, such partnerships must focus on specific areas, which will serve to improve access to appropriate antibiotics in LMICs. These areas include:

- **Building high-quality healthcare systems:** A high-quality healthcare system is defined as a health system that ‘optimizes health care in a given context, by consistently delivering care that improves or maintains health outcomes, by being valued and trusted by all people and by responding to changing population needs.’^175^ High-quality healthcare systems are: (i) people oriented—i.e. they are committed to patient safety, are accessible to patients and provide a conducive work environment for health workers to deliver high-quality health services;^175^ (ii) equitable—i.e. the provision of healthcare is devoid of disparities in the quality of health services rendered to individuals and groups in different socio-economic strata or diverse levels of underlying social disadvantage;^175^ (iii) resilient—i.e. health systems that adequately prepare for and respond to crises or emergencies and maintain core functions during crises. Resilient health systems are also adaptive health systems because they use insights and lessons learnt from such crises to reorganize and improve service delivery;^175,176^ and (iv) efficient—i.e. health systems that are prudent in the use of resources, avoid waste and achieve the maximum possible improvement in health outcomes with the investment received.^175^ In addition, high-quality healthcare systems guarantee healthcare as a fundamental human right, rather than a privilege based on an individual’s ability to afford healthcare.^177^ Such health systems are adequately funded and possess the required organization, technical expertise and infrastructure required to provide quality healthcare.^175^ In the absence of high-quality healthcare systems, efforts to improve access to appropriate antibiotics in LMICs will remain fragmented, unsustainable and fail to achieve the much needed benefit of simultaneously curbing AMR and improving access to appropriate antibiotics.^178^ Ultimately, access to appropriate antibiotics will require robust and efficient health services.^178^ Clearly, LMICs require support (funding, infrastructure, capacity building, etc.) to build high-quality healthcare systems that will guarantee access to quality healthcare, including access to antibiotics.^178^ Such support may come from strategic partnerships involving global organizations, HICs, financial institutions and the private sector.^178^ For example, global organizations could provide technical expertise or capacity building in CMS, AMS, procurement and drug quality testing, whereas HICs and financial institutions could provide funding to purchase diagnostic microbiology equipment.^178^
- **Streamlining bureaucratic protocols:** Another focus area of such partnerships will be to provide technical support to assist LMICs in streamlining bureaucratic protocols to facilitate seamless registration and entry of antibiotics.^6,12^ For example, the African Medicines Regulatory Harmonization (AMRH) programme was established in 2009 to solve the numerous challenges (such as inefficient medicine registration processes, poor regulatory frameworks, dearth of technical capacity, etc.) encountered by National Medicine Regulatory Authorities (NMRAs) in Africa.^179^ As mentioned earlier, such challenges delay registration of antibiotics, and also discourage pharmaceutical companies from initiating registration of antibiotics in LMICs.^6,12,179^ To overcome these challenges, the AMRH programme works with regional blocks of African NMRAs (such as the Economic Community of West African States, the East African Community and the Southern African Development Community) to harmonize medicine registration protocols and improve the quality of the registration process, thereby improving access to quality, safe, efficacious and affordable medicines in Africa.^179^ The AMRH programme is a shift from the current individual country/national registration of medicines to a simplified collaborative regional approach.^179–182^ To ensure effectiveness and efficiency, the AMRH programme works with partners under the aegis of a multi-stakeholder platform, the AMRH Partnership Platform (AMRH PP).^179^ The AMRH PP is an example of how strategic partnerships can support LMICs in efforts to improve access to appropriate antibiotics.^179^ Also, the proposed African Medicines Agency (AMA) will complement and enhance efforts of the AMRH programme by coordinating national and subregional medicine regulatory authorities to improve access to quality, safe and efficacious medicines, medical products and technologies in Africa.^83^
- **Building regulatory science capacity in LMICs:** It will also be necessary to build capacity in regulatory science in LMICs.^78–83^ In this regard, a major recommendation is to increase the number of training facilities and postgraduate programmes in regulatory sciences in LMICs.^78–83^ For example, the establishment of Regional Centres of Regulatory Excellence (RCOREs) by the African Union is a good development.^81^ The RCOREs possess expertise in the practice of regulatory science, and also provide training and capacity building.^81^ Adequate funds should, however, be made available to ensure these programmes (such as RCOREs) deliver quality education.^78–80^ In addition, to ensure equity and inclusiveness, scholarships and financial support should be made available to students.^78–80^ Furthermore, LMIC governments should streamline work processes in National Regulatory Authorities (NRAs), and ensure that the regulatory science profession has a clearly delineated career structure and progression.^78–80^ Also, job descriptions should be specific and clearly detail the required competencies and responsibilities of each regulatory science position in NRAs.^78–80^ In addition, work conditions should be improved to encourage staff recruitment and retention.^78–80^ Similarly (in accord with the multidisciplinary nature of regulatory science), it will be necessary to diversify the range of staff in NRAs beyond pharmacy and clinical sciences by recruiting professionals from relevant disciplines, such as engineering, law, economics and sociology.^78–80^
- **Developing and implementing NAPs on AMR:** LMIC governments will require support in developing and implementing NAPs on AMR, as the One health approach to NAPs on AMR calls for cost-intensive coordination and collaboration between human health, animal health, agriculture and food production sectors.^183^ For example, strategic partnerships could provide the needed funding and technical support required in prioritizing, budgeting for and implementing activities outlined in NAPs on AMR.^183^
- **Support in implementing AMS programmes:** AMS programmes are a key component of a quality healthcare system, as they ensure access to and rational use of appropriate antibiotics.^22^ Some LMIC governments will, however, require technical (e.g. capacity-building for developing prescribing guidelines, providing education to prescribers on appropriate antibiotic prescribing, etc.), financial (e.g. providing funds to support AMS activities such as educational programmes on appropriate prescribing) and infrastructural (e.g. providing diagnostic microbiology equipment) support to build quality AMS programmes that are integrated with other key health system components, including components required to improve access to appropriate antibiotics.^22,184^ In addition, integrating AMS programmes with the adoption of the WHO Essential Medicines List AWaRe (Access, Watch and Reserve) classification of commonly used antibiotics will also help to improve access to appropriate antibiotics in LMICs.^185–187^ This classification serves to monitor appropriate antibiotic use, improve access to antibiotics and curb AMR.^186,187^ The classification categorizes antibiotics into three groups: Access, Watch and Reserve. Access antibiotics (such as amoxicillin and ampicillin) represent first- or second-line antibiotic choices for empirical treatment of common infectious syndromes, based on a systematic assessment of the available evidence.^186,187^ They have a favourable safety profile and a low propensity to further aggravate AMR.^186,187^ Access antibiotics should be available at all times, at an affordable cost and of good quality (while still making efforts to ensure their appropriate use).^186,187^ Antibiotics in the Watch group (such as ceftazidime and ciprofloxacin) are prioritized as targets of stewardship programmes (and monitoring), and are recommended only for specific, limited indications as they present a greater potential to negatively impact AMR.^186,187^ Their use should be tightly monitored and restricted to the limited indications.^186,187^ The Reserve group of antibiotics (such as polymyxins and ceftazidime/avibactam) should only be used as a last resort when all other antibiotics have failed.^186,187^ They have activity against MDR or XDR bacteria, and therefore represent a valuable non-renewable resource that should be used as sparingly as possible.^186,187^ Their use should be closely monitored and prioritized, as targets of stewardship programmes, to ensure their continued effectiveness.^186,187^ When antibiotics in the Access group comprise at least 60% of national antibiotic consumption, this will result in better use of antibiotics, reduced costs and increased access to appropriate antibiotics.^186,187^
- **Developing, implementing and revising National Medicine Policies:** LMICs also need support to develop, implement and revise National Medicine or National Drug Policies.^188,189^ As defined by the WHO, a National Medicine Policy (NMP) is ‘a commitment to a goal and a guide for action. It expresses and prioritizes the medium- to long-term goals, set by the government, for the pharmaceutical sector, and identifies the main strategies for attaining them. It provides a framework within which the activities of the pharmaceutical sector can be coordinated. It covers both the public and the private sectors, and involves all the main actors in the pharmaceutical field.’^189^ Also, according to the WHO, an NMP serves as an LMIC government’s formal record of aspirations, aims, decisions and commitments in the pharmaceutical sector.^189^ Essentially, an NMP ensures: access (i.e. equitable availability and affordability) to essential medicines; quality, safety and efficacy of all medicines; and appropriate use of medicines by health professionals and consumers.^189^ By developing NMPs, LMIC governments have a broad overview of requirements in their pharmaceutical sectors, and are better positioned to streamline activities, and clearly define goals and responsibilities of all participants in their pharmaceutical sectors.^189^ In addition, the WHO recommends that all countries formulate and implement an NMP.^190^ However, several LMICs that have developed their NMPs have significant challenges with implementation.^191^ For example, a study showed that implementation challenges in the South African Development Community included: inadequate political will, poor understanding of NMPs by key stakeholders, a dearth of monitoring and evaluation, and a dearth of regular stakeholder engagement in developing, implementing and monitoring the progress of NMPs.^191^ In addition, the above-mentioned study showed that only 50% of countries had revised their NMPs within 10 years post-launch in the period 2011 to 2021.^191^ Clearly, LMICs will require the support of strategic partnerships to develop, implement and revise NMPs to ensure access to quality and affordable medicines, including appropriate antibiotics.^191,192^ For example, the WHO provides guidance and training (i.e. technical briefings, seminars) to support LMICs to develop and implement NMPs and strengthen their pharmaceutical sectors.^190^
- **Building efficient CMS:** LMIC governments will require support to build efficient CMS.^41–45,51^ Areas of support will include: developing and implementing National Laboratory Strategic Plans to address technical and infrastructural gaps in laboratory capacity; training and capacity development; provision of infrastructure, materials and equipment; and enrolment in quality assurance programmes.^41–45,51^
- **Boosting local antibiotic production:** As mentioned earlier, local antibiotic production is a major access strategy (to improve access to appropriate antibiotics) in LMICs.^6,12,96–99,108^ Clearly, LMICs will benefit from strategic partnerships geared towards boosting local antibiotic production.^193,194^ For example, in a similar vein to the AMRH programme’s regionally focused approach of medicine registration, the United Nations Conference on Trade and Development advocates for a regional approach (or regional antibiotic manufacturing hubs^12^) to local antibiotic production in Africa.^195^ The rationale for this is simple. A regional approach will harmonize registration procedures, expand markets, reduce production costs, and build regional value chains and business-to-business linkages that benefit all participant countries.^195^ For example, Nigeria has a population of over 200 million people and a larger market size than many of its neighbours in West Africa.^12,195^ Rather than establishing individual manufacturing plants in several West African countries, with smaller market sizes and demands, Nigeria could instead serve as a regional antibiotic manufacturing hub for countries in West Africa.^12^ Antibiotics produced in Nigeria could therefore serve the needs of the entire West African sub-region.^12^ In this regard, Ghana’s strategic partnership (with UNIDO, the European Union, the German Development Corporation and other strategic partners) to become a regional pharmaceutical manufacturing hub in sub-Saharan Africa, is commendable, and the nation must do everything possible to achieve and sustain this laudable initiative.^74^ Similarly, local antibiotic production can also be boosted by strategic initiatives involving LMIC governments (or local antibiotic manufacturers) and multinational pharmaceutical companies.^12^ Examples of such initiatives include: public private partnerships; technology transfers (i.e. a multinational pharmaceutical company shares specific knowledge about the process to make a particular antibiotic with a local pharmaceutical manufacturer);^12^ voluntary licensing agreements (a voluntary licence is an authorization given by a patent holder to a generic pharmaceutical manufacturer, allowing it to produce the patented medicine as if it were a generic and often at a lower cost than the patented version of the medicine);^12,196^ capacity building (i.e. providing equipment or training to employees of local pharmaceutical companies);^12^ and tax holidays (i.e. a ‘tax holiday’ allows a new company to operate for a specified number of years before paying corporate income taxes).^197^ For example, Novartis, a multinational pharmaceutical company, has partnered with local companies in Pakistan to transfer manufacturing knowledge and produce antibiotics locally.^198^ Similarly, the government of Bangladesh offers tax holidays to domestic manufacturers of APIs.^199^
- **Adopting innovative pricing strategies:** Pricing strategies are required to make appropriate antibiotics more affordable to patients in LMICs.^12^ Examples of such pricing strategies include: price caps (i.e. an LMIC government and a pharmaceutical company agree on the maximum selling price for an antibiotic);^12^ tiered or differential pricing (i.e. individuals in LMICs pay lower prices for the same antibiotic than individuals in HICs);^12^ equitable pricing policies (i.e. prices of antibiotics are based on the purchasing power of an LMIC’s population);^12^ patient assistance initiatives (i.e. pharmaceutical companies provide free or highly subsidized antibiotics to specific patient groups who cannot afford such antibiotics);^12^ Early Access Programmes (i.e. these programmes allow patients to access yet-to-be-registered generic or patented antibiotics);^12^ and donations of antibiotics to an LMIC (e.g. during national emergencies such as epidemics, war, natural disasters, etc.).^12^ The aforementioned donation of azithromycin (by the manufacturer) to trachoma-endemic communities is an example of a pricing strategy. In the absence of donations from the manufacturer, the antibiotic (i.e. azithromycin) would be unaffordable to patients in these communities.^51^ Similarly, the WHO has developed a guideline to assist countries in managing pharmaceutical prices.^200^ In addition, the WHO also has training workshops on pricing strategies for country stakeholders and an annual stakeholder engagement forum (i.e. the Fair Pricing Forum) to enable discussion and information sharing on appropriate pricing strategies and policies.^200–202^ According to the WHO, a ‘fair price’ implies that a medicine is ‘affordable for health systems and patients, and that at the same time provides sufficient market incentive for industry to invest in innovation and the production of medicines. In other words, fairness here implies positive incentives and/or benefits for all stakeholders—i.e. those who purchase and use medicines, and those involved in the R&D and manufacture of medicines.’^201^
- **Building efficient procurement and SCM systems:** These partnerships could support LMICs in participating in tenders to secure quantities of antibiotics that can meet a country’s needs at affordable prices.^12^ In addition, a pooled procurement process can occur in which the partnership supports several LMIC governments to pool funds and order antibiotics.^12^ Also, a global procurement hub could be created.^203^ The hub could be funded by donor agencies and HICs and would be responsible for registration, procurement, distribution, stewardship, and providing data on antibiotic consumption and resistance.^203^ The hub would therefore aggregate demand for antibiotics (such as antibiotics with supply chain problems), reduce market failures and make the whole market more robust.^203^ For example the SECURE programme, a joint effort by the WHO and the Global Antibiotic Research and Development Partnership (GARDP), seeks to improve access to antibiotics in LMICs through pooled procurement mechanisms, by aggregating demand while ensuring appropriate antibiotic use.^204^ Also, robust drug SCM systems for existing programmes (e.g. HIV, TB and malaria) could be expanded to include antibiotics, particularly to vulnerable patient groups such as individuals in far-flung rural areas with poor road networks.^3,6^ Similarly, adopting alternative means of transportation, such as drones, could help in supplying antibiotics to health facilities in remote areas.^205^
- **Combating the menace of substandard and falsified antibiotics**. Such partnerships can assist LMIC governments in tackling the problem of substandard and falsified antibiotics.^206^ For example, the WHO Member State Mechanism on Substandard and Falsified Medical Products is a collaborative network among WHO member states that seeks to ‘promote access to affordable, safe, efficacious, and quality medical products, and to promote through effective collaboration among Member States and the Secretariat, the prevention and control of substandard and falsified medical products, and associated activities.’^206^ In addition, pharmaceutical companies can participate in quality assurance processes, such as the WHO prequalification of medicines programme, which allows medicines to be purchased by or through international procurement agencies without registration, making them more easily available to patients in LMICs.^207^ Similarly, the Promoting the Quality of Medicines (PQM) programme is a United States Agency for International Development (USAID) programme that supports medicine quality assurance processes in LMICs.^208^ The programme works with: national medicines regulatory authorities (to strengthen quality assurance and quality control systems and implement risk-based approaches to optimize the regulation of medicines); quality control laboratories (to improve local capacity to accurately and reliably test the quality of medicines); and manufacturers (to increase the supply of quality-assured medicines that comply with internationally recognized good manufacturing practices and standards).^208^ Similarly, the AMA is expected to contribute to increased availability of affordable and acceptable (quality, safety, efficacious) medical products on the African continent, and reduce the incidence of substandard and falsified medicines.^83^
- **Improving surveillance and data collection systems:** Improved surveillance and data collection will promote knowledge of local resistance patterns, and therefore help to guide procurement of antibiotics at health facility and national levels.^6,12,51^ LMIC governments will require support in building effective surveillance and data collection systems.^6,12,51^ Through strategic partnerships, LMIC governments can be empowered to improve local diagnostic microbiology capacity, and increase the number and capacity of AMR surveillance sentinel sites.^6,12,51^ In addition, LMIC governments should scale up the participation of laboratories in quality assurance programmes, with a view to establishing robust quality assurance processes in diagnostic microbiology laboratories.^6,12,51^ Also, the adoption of globally accepted platforms for surveillance of AMR and antibiotic consumption will help in building robust surveillance systems.^209–211^ Examples of these platforms include WHONET, the Global Antimicrobial Resistance Surveillance System (GLASS) and the Global Point Prevalence Survey (GPPS).^209–211^ WHONET supports laboratory-based surveillance of AMR.^209^ GLASS is an AMR surveillance system that includes epidemiological, clinical and population-level data.^210^ GPPS is a global surveillance system that measures antimicrobial consumption and resistance in participating hospitals.^211^
- **Supporting LMICs to curb poverty, unemployment and illiteracy:** Although a full discussion on efforts to curb poverty, unemployment and illiteracy is clearly outside this review’s focus, it is still important to note that poverty eradication, job creation and curbing illiteracy have a net benefit of improving an individual’s quality of life and empowering individuals to access healthcare (and ultimately access to appropriate antibiotics).^212,213^ Since access to health services in many LMICs is out-of-pocket, poverty and unemployment therefore deny many individuals access to healthcare, including appropriate antibiotics.^6,214^ The related problem of illiteracy means that LMIC individuals who are not literate are unable to make informed choices regarding their health, including the appropriate use of antibiotics.^6,215^ For example, an illiterate patient may not clearly understand the explanation of a prescriber or dispenser who is speaking English, or who cannot speak the native dialect of the patient.^216^ In addition, an illiterate individual may not understand basic health information about the appropriate use of antibiotics (such as dosage instructions on a drug leaflet or package that are written in English) or the need to avoid obtaining antibiotics from unlicensed vendors.^216^ Even when a translator/interpreter is used there is a danger of misinterpretation, particularly if the translator/interpreter is not fluent in the patient’s language.^216^ Therefore efforts to address antibiotic resistance must occur simultaneously with efforts to eradicate poverty, create jobs, provide education, housing, and facilities for water, sanitation and hygiene.^212,213^ Also, pharmaceutical companies should adopt multilingual labelling/packaging particularly in local LMIC languages.^217,218^ Multilingual packaging is the use of two or more languages for at least one component of the packaging material (such as the package leaflet or the outer packaging) of a pharmaceutical or medicinal product.^217^ Labelling in a local language promotes the safe (or appropriate) use of medicines, as it makes it possible for individuals (who cannot read English) to easily read and understand instructions in the medicine’s leaflet. This (i.e. labelling a medicine in a local language) is better than relying on pharmacists or drug vendors, who may not have the time to explain or even the capacity to correctly translate labels or instructions in English into a local language.^218^
- **Incentivize R&D for new and effective antibiotics and include LMICs in the antibiotic R&D pipeline:** A major response to dwindling R&D efforts would be to provide incentives to boost R&D of new and effective antibiotics.^68,219–222^ These incentives include: push, pull and hybrid incentives.^219,220^
- ***Push incentives*** provide funding to support innovation, research and development of new antibiotics, from the early stages of basic science to clinical trials, regardless of successful access to the market.^219–222^ They therefore lower the economic risk associated with the failure of a potential drug candidate.^219–222^ Examples of push incentives include: increasing access to research; providing research grants; technical support; offering tax incentives or waivers; and establishing public-private partnerships for sharing R&D outlays.^219–222^ For example, CARB-X funds the development of antibiotics (and other life-saving products such as vaccines and rapid diagnostics).^127,223^ Another example of a push incentive is the Innovative Health Initiative (i.e. IHI, originally known as the Innovative Medicines Initiative until 2021, when it transitioned to the IHI).^224,225^ The IHI is a public-private partnership between the European Union and European life science industries that funds antibiotic development through innovative programmes.^224,225^ One of these innovative programmes is the New Drugs 4 Bad Bugs (ND4BB) programme, a €650 million programme comprising eight projects finding solutions to the scientific, regulatory and business challenges that hamper the development of new antibiotics.^225^ Another example of a push incentive is the Joint Programming Initiative on Antimicrobial Resistance (i.e. JPIAMR). JPIAMR is an international collaborative platform that coordinates national research funding and supports collaborative action for filling knowledge gaps on AMR with a One Health perspective.^226^ Also, the US Center for the Biomedical Advanced Research and Development Authority’s (i.e. BARDA) Antibacterials programme provides support to antibiotic developers.^227^ This support spans the development pipeline, ranging from preclinical and clinical research through regulatory approval and, ultimately, procurement for national preparedness.^227^ The BARDA Antibacterials programme has invested over $1.6 billion in more than 120 antibacterial development products primarily via three programme areas: the CARB-X programme; the Advanced Research and Development portfolio; and Project BioShield. Examples of some approved products include: Varbomere (i.e. meropenem/vaborbactam) and Xerava (i.e. eravacycline).^227^
- ***Pull incentives*** reward successful antibiotic development and also raise the net present value (NPV) of novel or newly developed antibiotics.^219–222,228^ The NPV describes the relationship between project costs (i.e. the costs of developing an antibiotic) and revenue in terms of discounted cash flow.^221,222,228^ The NPV is therefore the sum of all investment costs from antibiotic development and expected present value of future revenues, considering the discounted rate of the time value of money for a given development programme.^221,222,228^ The NPV is used by the pharmaceutical industry to estimate the risk/benefit and profitability of investing in developing a new pharmaceutical product, such as a new antibiotic.^221,222,228^ A low NPV will therefore discourage investment.^221,222,228^ Typically, the NPV for antibiotics is approximately US$42.61 million.^221,222,228^ This value is low compared with the NPV for neurological or musculoskeletal drugs, which ranges from US$720 million to US$1.15 billion.^221,222,228^ To incentivize antibiotic development, an NPV of about US$200 million is suggested.^221,222,228^ Clearly, pull incentives are crucial for antibiotic development as (in addition to raising the NPV of new antibiotics) they ensure profitability, adequate market revenue, and reduce the risk of market failures arising from low sales (and insufficient revenues) from newly approved antibiotics.^219–222,228^ Pull incentives are divided into outcome based and lego-regulatory incentives.^219,221^
- Outcome-based pull incentives provide rewards to developers of newly approved antibiotics.^219,221^ Examples of outcome-based pull incentives include market entry rewards (i.e. monetary rewards provided to developers following successful market entry of a new antibiotic), patent buyouts (i.e. an antibiotic developer is offered a large sum of money in exchange for the intellectual property rights of a successfully developed antibiotic) and delinkage models.^219,221^ The delinkage model seeks to ‘delink’ or separate profit of antibiotics from the number of units sold, and therefore guarantees return on investments (for pharmaceutical companies) well above what could be obtained from regular sales.^229–232^ The delinkage model involves payment for drug innovation, based on public health value, rather than on a per-use basis.^229–232^ An example of a delinkage model is the proposed Pioneering Antimicrobial Subscriptions to End Upsurging Resistance (PASTEUR) Act.^233,234^ Through this model the US government will make regular payments (i.e. subscriptions) for novel antibiotics, thereby guaranteeing patients on government programmes free access to these antibiotics.^231,233,234^ Similarly, the UK has deployed the delinkage model to make annual subscriptions to purchase two reserve antibiotics (cefiderocol and ceftazidime/avibactam) for the NHS.^231,235,236^ These payments are made irrespective of quantities of these antibiotics consumed by the NHS.^231,235,236^ As a result, NHS patients have access to these antibiotics, and simultaneously the manufacturers of these antibiotics make profits.^231,235,236^ Certainly, this is a win-win situation for NHS patients, the UK government and pharmaceutical companies.^231,235,236^ In this regard a global delinkage model is a possible way of ensuring access to appropriate antibiotics in LMICs.^229,232^ By pooling resources together, strategic partnerships (involving donor organizations, HICs and LMIC governments) could finance global delinkage models that would pay annual subscriptions to specific pharmaceutical companies for much-needed antibiotics in LMICs.^229,232^ LMICs that are unable to contribute financially to such partnerships could make commitments to appropriate use and surveillance of such antibiotics.^229,232^
- Lego-regulatory pull incentives create policies that accelerate the market approval process for newly approved antibiotics, extend market exclusivity rights and increase reimbursement prices.^219–222^ For example, the Generating Antibiotic Incentives Now (GAIN) Act is a US bill, ratified in 2012, that provides fast-track review (and approval) and 5 years of additional market exclusivity for an antimicrobial designated by the US FDA as a ‘qualified infectious disease product’ (QIDP).^219,237^ A QIDP is an antimicrobial agent used to treat serious or life-threatening infections caused by antibacterial- or antifungal-resistant pathogens, including novel or emerging infectious pathogens.^237^ Examples of antimicrobial agents that have received GAIN exclusivity include: ceftolozane/tazobactam,^238^ imipenem/cilastatin/relebactam^239^ and dalbavancin.^240^
- ***Hybrid incentives*** are a mix of push and pull incentives.^219^ Clearly push and pull mechanisms have their advantages and disadvantages, and adopting a sole (i.e. push or pull) incentive may not be adequate.^219^ For example, push incentives reward early stages of antibiotic development; however, following approval and market entry, insufficient sales of a new antibiotic will ultimately result in market failure and bankruptcy of an R&D company.^219^ Therefore, hybrid strategies combine the benefits of both push and pull strategies to improve antibiotic R&D.^219^ Examples of hybrid strategies include the Office of Health Economics model,^241^ the Limited Population Pathway for Antibacterial and Antifungal Drugs (LPAD); and the Options Market for Antibiotics.^242^ For example, pretomanid tablets in combination with linezolid and bedaquiline have been approved under the LPAD pathway for the treatment of MDR and XDR TB.^242^ Another drug approved under the LPAD pathway is Arikayce (an amikacin liposomal inhalational suspension) for treating pulmonary disease caused by *Mycobacterium avium* complex bacteria that are non-responsive to conventional treatment protocols (i.e. refractory disease).^242^ In addition, global initiatives such as GARDP^68^ and the Antimicrobial Resistance Action Fund^243^ are examples of efforts incorporating both push and pull initiatives to ensure antibiotic R&D meets the needs of LMICs with high infectious disease burdens and high rates of AMR.^68,243^
- It is strongly recommended that incentives to boost R&D of new and effective antibiotics be extended to LMICs.^244–246^ New antibiotics from R&D in HICs hardly reach the shores of LMICs.^244^ Therefore a paradigm shift, characterized by antibiotic R&D in LMICs (and designed to meet the specific needs of LMIC populations), is required.^244–246^ However, LMICs will require support to participate in antibiotic R&D. Areas of support will include: streamlining regulations (such as regulations and protocols for clinical trials); funding and incentivizing antibiotic R&D; and capacity building.^244–246^ For example, through the support of strategic partnerships the AMA will be in a suitable position to coordinate antibiotic R&D in Africa by: providing a suitable regulatory environment to foster R&D on the continent; strengthening existing R&D platforms (such as the Pan African Clinical Trials Registry^247^); leveraging partnerships with HICs and donor organizations to expand funding calls to the continent; and building capacity in African nations to participate in antibiotic R&D.^83,244–246^ In addition, it is recommended that stakeholders (such as global organizations, civil society organizations, professional bodies, policy entrepreneurs, etc.) begin drawing attention to including LMICs in antibiotic R&D.^244–246^

### Conclusions

Inequitable access to appropriate antibiotics in LMICs is associated with poor treatment outcomes and the emergence of AMR.^6,7,10–15^ Whereas increased political commitment by LMIC governments is a key factor required to improve access to antibiotics in LMICs, many LMICs lack the required technical, infrastructural and financial ability to improve access to appropriate antibiotics.^6,12,21,39,73,75,76,146^ LMIC governments will therefore require support to improve access to appropriate antibiotics.^6,12,21,39,73,75,76,146^ Such support could occur through strategic partnerships involving LMIC governments, HIC governments, global organizations and pharmaceutical companies.^6,12,21,39,73,75,76,146^ In particular, efforts to streamline bureaucratic processes and improve the registration and entry of appropriate antibiotics into LMICs are required.^6,12,180^ For example, the AMRH programme and the AMA are two initiatives that have immense potential to streamline bureaucratic processes, and increase access to appropriate antibiotics in Africa.^83,180^ In addition, the dwindling antibiotic pipeline further reduces the arsenal of effective antibiotics, particularly in LMICs.^6,12,89,113,114^ In this regard holistic strategies and incentives that support early and later stages of antibiotic development (including market entry) are required to incentivize the development of new and effective antibiotics and ensure that these antibiotics are available to patients in LMICs who require them.^219–222^ However, LMICs should also be included in these incentives through increased participation in research, development and clinical trials.^244–246^ There is a dire need for a paradigm shift from the current model of developing new antibiotics in HICs and then attempting to introduce them to LMICs.^244^ This model is broken, and has failed to ensure access to new and effective antibiotics in LMICs.^244^ Instead, it is recommended that LMICs should be supported to become antibiotic R&D hubs, and produce new and effective antibiotics that would meet the specific needs of their populations.^244–247^
